# Association of a low ankle brachial index with progression to end-stage kidney disease in patients with advanced-stage diabetic kidney disease

**DOI:** 10.1080/0886022X.2022.2160347

**Published:** 2023-01-12

**Authors:** Ruiying Tang, Yun Liu, Jiexin Chen, Jihong Deng, Yan Liu, Qingdong Xu

**Affiliations:** a Department of Nephrology, Jiangmen Central Hospital, Jiangmen City, China; b Department of Nephrology, Guangzhou Red Cross Hospital, Jinan University, Guangzhou City, China

**Keywords:** Ankle-brachial index, end-stage kidney disease, diabetes, diabetic kidney disease

## Abstract

**Introductions:**

The effect of a low ankle-brachial index (ABI) in patients with advanced-stage diabetic kidney disease is not fully understood. This study investigates the prevalence of a low ABI in patients with advanced-stage diabetic kidney disease, which was defined as a urinary albumin-to-creatinine ratio (UACR) ≥300 mg/g and an estimated glomerular filtration rate (eGFR) between 15–60 mL/min/1.73 m^2^. Furthermore, the association between a low ABI and end-stage kidney disease (ESKD) was determined.

**Methods:**

This single-center, retrospective, cohort study included 529 patients with advanced-stage diabetic kidney disease who were stratified into groups according to the ABI: high (>1.3), normal (0.9–1.3), and low (<0.9). The Kaplan-Meier method and Cox proportional analysis were used to examine the association between the ABI and ESKD.

**Results:**

A total of 42.5% of patients with a low ABI progressed to ESKD. A low ABI was associated with a greater risk of ESKD (hazard ratio (HR): 1.073). After adjusting for traditional chronic kidney disease risk factors, a low ABI remained associated with a greater risk of ESKD (HR: 1.758; 95% confidence interval: 1.243–2.487; *p* = 0.001).

**Conclusions:**

These results indicate that patients with a low ABI should be monitored carefully. Furthermore, preventive therapy should be considered to improve the long-term kidney survival of patients with residual kidney function.

## Introduction

In 2017, 451 million patients had type 2 diabetes mellitus (DM) worldwide, and this number is expected to increase to 693 million by 2045 [1]. The urinary albumin-to-creatinine ratio (UACR) and estimated glomerular filtration rate (eGFR) are commonly used as screening parameters and clinical staging criteria for diabetic nephropathy [2], a microvascular complication of DM. As the leading cause of hemodialysis in developed countries, diabetic nephropathy occurs in approximately 35% of patients with DM and is associated with adverse clinical outcomes [3,4].

The ankle-brachial index (ABI) has recently become a routine screening parameter for vascular complications in patients with DM [5]. A correlation between the ABI and microvascular complications in diabetes has been widely reported [6]. The ABI is calculated as the ankle-to-arm systolic blood pressure (SBP) ratio. It is a simple, noninvasive screening tool used to detect peripheral arterial disease (PAD) [7,8] as it reflects the aging and pathological state of blood vessels. An ABI threshold of 0.90 has been reported to have 90% sensitivity and specificity to detect PAD when compared to angiography methods [9]. A low ABI (<0.9) is a predictor of cardiovascular disease, stroke, and mortality in the general population and in patients with DM and chronic kidney disease (CKD) [10–13]. Atherosclerosis also contributes to the deterioration of kidney function, as a low ABI is predictive of future diminished kidney function and is associated with an increased risk of CKD and decreased eGFR [14–16]. Additionally, a close relationship between low ABI and early-stage CKD was found in patients with diabetes with normal albuminuria [15], suggesting that a low ABI level contributed to diminished kidney function independent of albuminuria. However, U-shape relationships between the ABI and eGFR, CKD, cardiovascular disease, and all-cause mortality have also been reported [9,17].

To the best of our knowledge, no study regarding the association between the ABI and the progression to end-stage kidney disease (ESKD) in patients with advanced-stage diabetic kidney disease has been reported. Therefore, this single-center, retrospective, cohort study determines the effect of the ABI on adverse kidney outcomes and progression to ESKD in patients with advanced-stage diabetic kidney disease, providing a theoretical basis for its potential clinical role.

## Materials and methods

### Patients

This single-center, retrospective, cohort study included 529 patients with type 2 DM and advanced-stage diabetic kidney disease. Patients ≥18 years of age with type 2 diabetic kidney disease, an UACR ≥300 mg/g, and an eGFR between 15–60 mL/min/1.73 m^2^ who did not undergo kidney replacement therapy and for whom ABI data were available were included in this study. Patients with a baseline eGFR <15 mL/min/1.73 m^2^ and those with acute infections (such as pneumonia, diarrhea, cholecystitis, or end-stage cancers) were excluded from the study. The study was approved by the investigational review board of Jiangmen Central Hospital (approval number 2022103) and was conducted in accordance with the Declaration of Helsinki.

### Clinical data

The patients’ baseline characteristics, including age, sex, DM duration, height, weight, medication list, and blood pressure were obtained from the medical records.

Data on the white blood cell count (WBC), hemoglobin level (HGB), platelet count, neutrophil count, 24-h proteinuria, UACR, liver function tests, kidney function tests, blood glucose level, glycated hemoglobin (HbA1C) level, serum albumin (ALB) level, blood lipid levels, eGFR, parathyroid hormone level, C-reactive protein (CRP) level, and uric acid (UA) level were also obtained.

### ABI measurement

The ABI was measured with the patient in the supine position on the same day that the patient’s kidney function was evaluated. The blood pressure was measured at the bilateral brachial and ankle arteries using an arteriosclerosis Doppler monitor (OMROM, HXV-ST 1, China). According to the American Heart Association guidelines [18], systolic blood pressure is measured in each arm and in the dorsalis pedis and posterior tibial arteries in each ankle. The higher of the two arm pressures is selected, as is the higher of the two pressures in each ankle. The right and left ABI values are determined by dividing the higher ankle pressure in each leg by the higher arm pressure and the average of the right and left ABI was used for the analysis. In this study, the ABI ranged from 0.32 to 1.96; therefore, patients were categorized into three groups based on the ABI: high (ABI > 1.3), normal (0.9 ≤ ABI ≤ 1.3), and low (ABI < 0.9).

### Calculation of eGFR

The eGFR from serum Creatinine was estimated using the modification of diet in kidney disease equation [19] (CKD-EPI; MDRD Study): eGFR = 186 × serum creatinine^−1.154^ × age^−0.203^ × 0.742 (females) × 1.233.

### Follow-up and study endpoint

The patients were followed up until December 31, 2021. The study endpoint was progression to ESKD, defined as an eGFR <15 mL/min/1.73 m^2^, or undergoing kidney replacement therapy.

### Statistical analysis

Normally distributed, continuous variables are presented as mean ± standard deviation. Continuous variables with a non-normal distribution are presented as median (interquartile range [IQR]). Differences among the three groups were assessed using one-way analysis of variance, the Kruskal–Wallis test, or the chi-squared test, as appropriate. The cumulative, event-free probabilities of ESKD were determined using the Kaplan-Meier method and compared using the log-rank test. A univariate Cox proportional analysis was conducted, and the relevant variables were included in a multivariate Cox proportional analysis using different models. The results are expressed as hazard ratios (HRs) with 95% confidence intervals (CIs). SPSS statistical software, version 22 (IBM Corp, Armonk, NY, USA), was used to conduct the statistical analyses. Statistical significance was set at *p* < 0.05.

## Results

**Figure 1.**
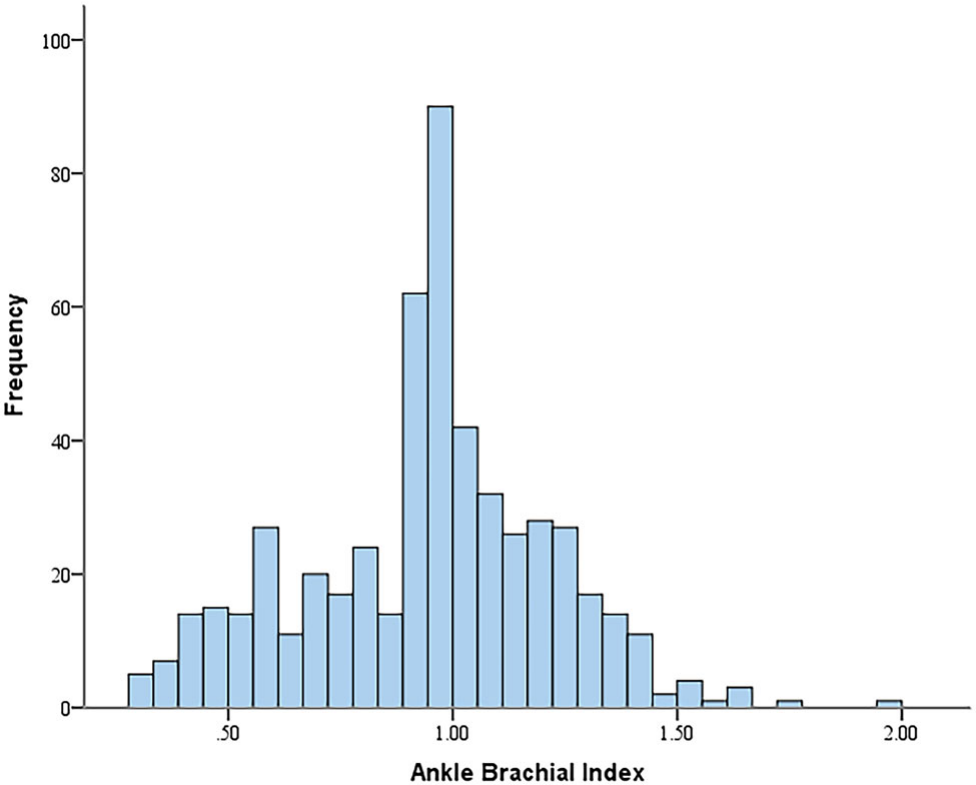
Distribution of ankle brachial index (ABI) levels.

A total of 529 individuals with advanced-stage diabetic kidney disease were followed up for a duration of 63.5 months (IQR: 37.7–67.1 months). The baseline ABI showed a non-normal distribution (Figure 1). During the follow-up period, 31.0% (164/529) of the patients developed ESKD, including 42.5% (74/174) in the low ABI group, 24.8% (76/306) in the normal ABI group, and 28.5% (14/49) in the high ABI group.

### Comparisons of clinical parameters between the groups

The baseline characteristics of the total study population and after stratification by the upper and lower limits of the normal range of ABI are presented in Table 1. Patients with a lower ABI were more likely to be female, older, and smokers, and had a higher SBP, serum CRP level, and serum total cholesterol (TC) level (all *p* < 0.05) (Table 1).

**Table 1.** Baseline demographic and clinical characteristics of patients with advanced-stage diabetic kidney disease categorized according to the ankle brachial index (ABI).

Variables	Total (*n* = 529)	0.9 ≤ ABI ≤ 1.3 (*n* = 306)	ABI < 0.9 (*n* = 174)	ABI > 1.3 (*n* = 49)	*p*
Age(years)	60.0 (51.0, 67.0)	59.0 (51.0, 66.0)	61.0 (51.0, 69.8)	59.0 (43.0, 65.0)	0.047*
Gender, female (%)	240 (45.4)	125 (40.8)	91 (52.3)	24 (49)	0.046*
Diabetic duration, (years)	4.0 (1.0, 9.0)	4.0 (1.0, 9.0)	5.0 (1.5, 10.0)	3.0 (1.5, 6.0)	0.089
Smoking, *n* (%)	125 (23.6)	59 (19.3)	63 (36.2)	3 (6.1)	<0.001*
Drinking alcohol, *n* (%)	80 (15.1)	44 (14.4)	29 (16.7)	7 (14.3)	0.786
Insulin, *n* (%)	271 (51.2)	155 (50.7)	86 (49.4)	30 (61.2)	0.328
Hypoglycemic drugs, *n* (%)	436 (82.4)	247 (80.7)	149 (85.6)	40 (81.6)	0.392
Statin, *n* (%)	275 (52.0)	157 (51.3)	93 (53.4)	25 (51)	0.894
Aspirin, *n* (%)	253 (47.8)	137 (44.8)	94 (54)	22 (44.9)	0.136
ACEI or ARB, *n* (%)	266 (50.3)	140 (45.8)	98 (56.3)	28 (57.1)	0.05
BMI (kg/m^2^)	23.8 (21.8, 26.4)	24.1 (21.9, 26.6)	23.5 (21.6, 25.5)	24.2 (22.0, 27.6)	0.148
SBP(mmhg)	137.0 (123.0, 150.0)	136.0 (123.0, 149.0)	141.0 (126.2, 152.0)	128.0 (120.0, 144.0)	0.009*
DBP (mmhg)	78.0 (69.0, 85.0)	77.0 (69.0, 85.0)	78.0 (67.2, 85.0)	78.0 (70.0, 86.0)	0.586
24 hours proteinuria(mg)	2665.0 (1375.0, 4600.0)	2839.0 (1410.0, 4916.0)	2402.0 (1232.0, 4084.0)	2941.0 (1769.0, 4755.0)	0.095
UACR, (mg/g)	1300 (700, 2300)	1400 (700, 2500)	1200 (600, 2000)	1500 (900, 2400)	0.1
HbA1C (%)	7.5 (6.5, 8.6)	7.4 (6.4, 8.6)	7.5 (6.5, 8.8)	7.8 (6.3, 8.6)	0.747
Glu (mmol/L)	7.8 (6.2, 9.0)	7.7 (6.1, 8.9)	7.8 (6.4, 9.1)	7.5 (6.6, 9.2)	0.734
sCR (umol/L)	124.0 (104.6, 147.6)	125.1 (105.2, 148.6)	122.5 (103.9, 145.8)	121.4 (104.8, 149.2)	0.888
eGFR(mL/(min·1. 73 m^2^ ))	38.8 (36.4, 41.6)	38.9 (36.3, 42.0)	38.6 (36.4, 40.9)	38.6 (36.8, 42.2)	0.457
HGB (g/L)	92.9 (88.7, 96.4)	92.5 (89.1, 96.3)	93.2 (88.6, 97.1)	93.1 (88.6, 95.4)	0.673
WBC (10^9^/L)	7.2 (6.0, 9.2)	7.1 (6.0, 9.1)	7.4 (5.9, 9.2)	7.6 (6.3, 9.5)	0.773
PLT(10^9^)	222.7 (181.5, 268.2)	220.2 (182.1, 264.8)	222.5 (179.0, 266.4)	227.1 (167.0, 283.0)	0.868
ALB (g/L)	33.7 (30.1, 36.5)	33.6 (30.3, 36.0)	34.1 (29.8, 37.4)	32.7 (30.4, 36.2)	0.68
TC (mmol/L)	5.4 (4.4, 6.2)	5.3 (4.4, 6.1)	5.7 (4.6, 6.7)	5.1 (4.2, 6.0)	0.015*
TG(mmol/L)	1.9 (1.2, 2.7)	1.9 (1.3, 2.6)	1.9 (1.1, 2.8)	1.7 (1.1, 2.6)	0.672
ALT (U/L)	20.0 (13.0, 33.0)	20.0 (13.8, 31.0)	21.0 (13.0, 35.0)	20.5 (13.0, 40.5)	0.852
AST (U/L)	26.0 (21.0, 34.0)	25.0 (21.0, 33.0)	27.0 (22.0, 34.0)	24.5 (20.2, 33.8)	0.389
ALP (U/L)	79.0 (64.0, 94.0)	81.0 (64.5, 95.5)	76.0 (63.5, 92.0)	75.0 (64.0, 93.8)	0.455
UA(mmol/L)	368.2 (286.8, 485.8)	371.0 (289.0, 490.9)	366.2 (284.0, 480.7)	369.3 (300.2, 485.6)	0.812
BUN(mmol/L)	10.4 (8.5, 13.4)	10.4 (8.2, 13.4)	10.5 (8.6, 13.2)	10.0 (8.6, 14.0)	0.503
CRP (mg/l)	1.7 (0.7, 3.3)	1.5 (0.5, 3.0)	1.9 (1.1, 3.7)	1.8 (0.8, 2.9)	0.008*

Abbreviations: ACEI: angiotensin-converting enzyme inhibitor; ARB: angiotensin receptor blocker; BMI: body mass index; SBP: systolic blood pressure; DBP: diastolic blood pressure; UACR: urinary albumin-to-creatinine ratio; HbA1C: glycated hemoglobin; Glu: serum glucose; sCr: serum creatinine; eGFR: glomerular filtration rate; HGB: hemoglobin; WBC: white blood cell; PLT: platelet; ALB: serum albumin; TC: total cholesterol; TG: triglycerides; UA: uric acid; BUN: serum urea nitrogen; CRP: C-reactive protein. *p < 0.05.

### Association between the ABI and ESKD in advanced-stage diabetic kidney disease

**Figure 2.**
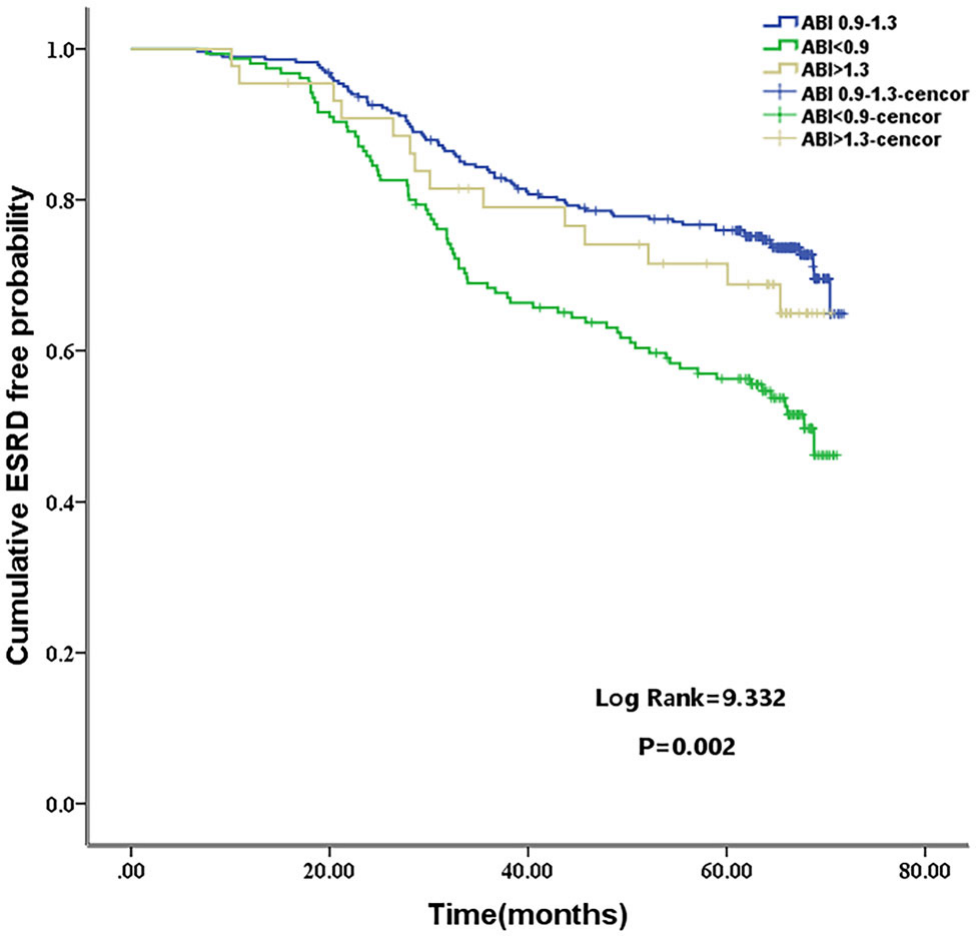
Cumulative survival curves for end-stage kidney disease (ESKD) according to the categorization of the ankle brachial index (ABI) levels at baseline.

A total of 164 patients developed ESKD events that were recorded during follow-up. The results of survival analysis for ESKD showed that ABI level <0.9 had the worst prognosis. Patients in the low ABI group had a significantly higher rate of ESKD events than patients in the other groups (ABI <0.9 vs 0.9 ≤ ABI ≤1.3 vs ABI >1.3; 42.5% vs. 24.8% vs. 28.5%, respectively; *p* = 0.002, log-rank test; Figure 2).

**Figure 3.**
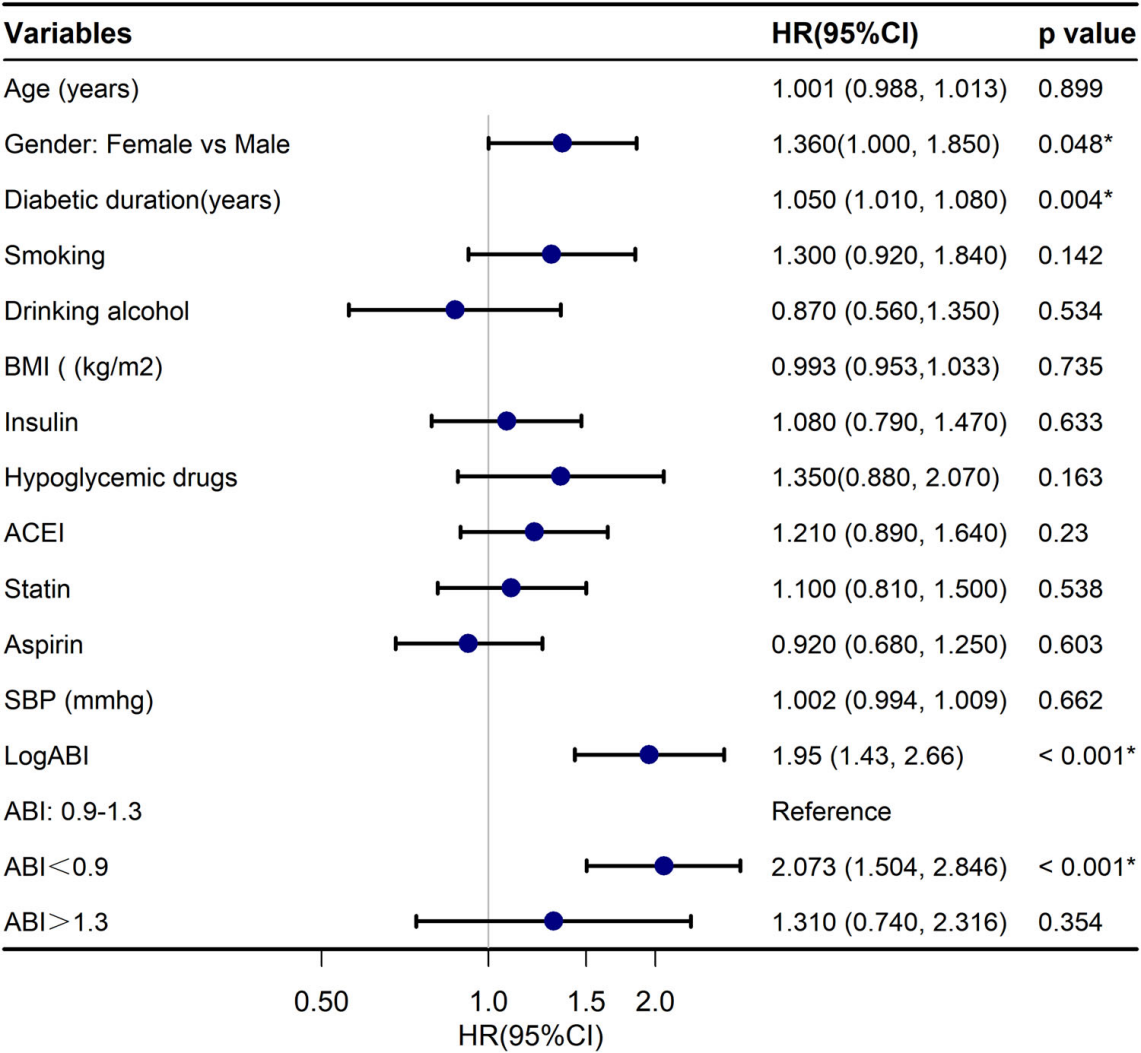
Crude hazard ratios for end-stage kidney disease (ESKD) by demographic variables. HR: hazard ratio; 95% CI: 95% confidence interval; BMI: body mass index; ACEI: angiotensin-converting enzyme inhibitor; SBP: systolic blood pressure; ABI: ankle brachial index.

**Figure 4.**
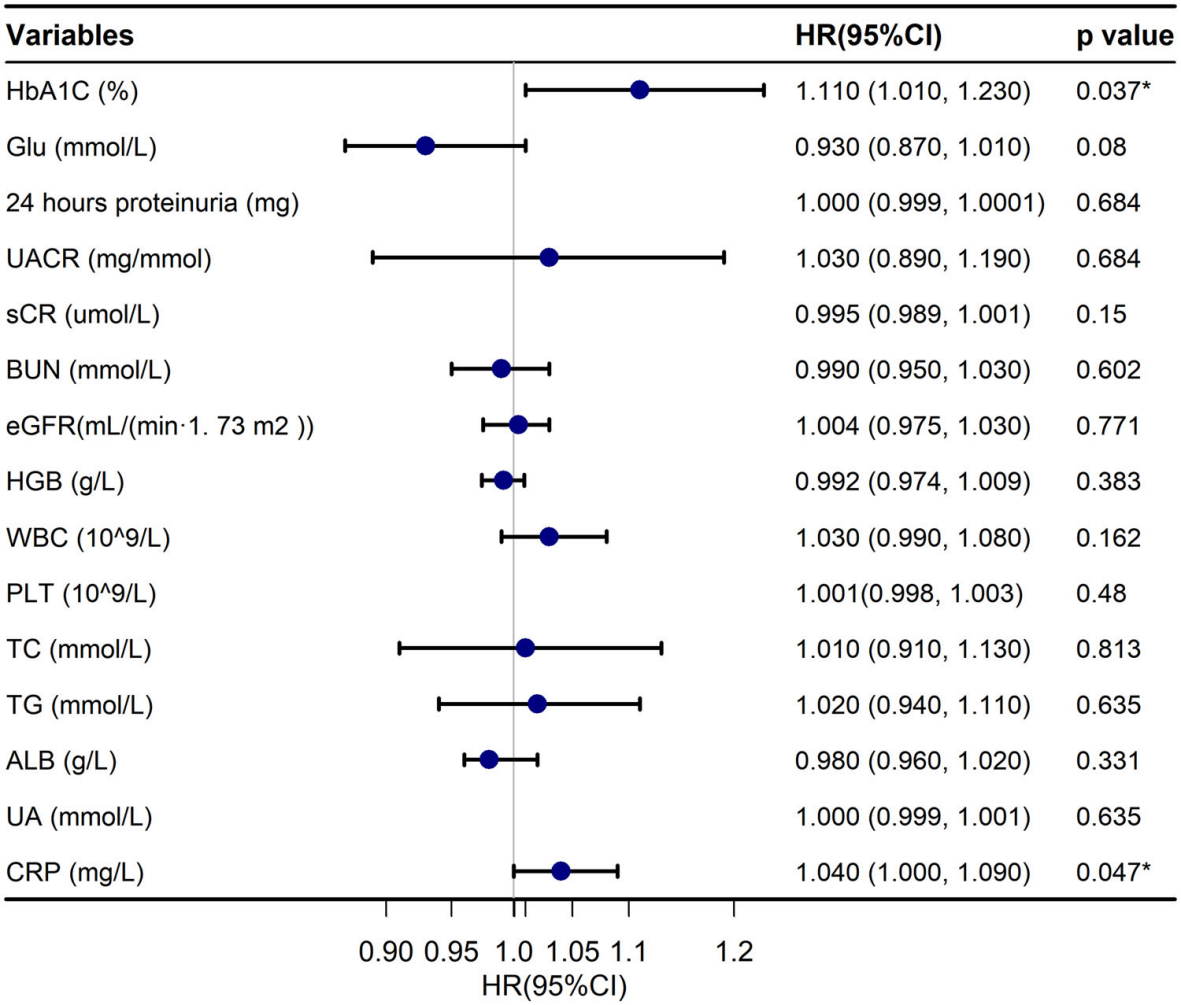
Crude hazard ratios for end-stage kidney disease (ESKD) by laboratory examination. HR: hazard ratio; 95% CI: 95% confidence interval; HbA1C: glycated hemoglobin; Glu: serum glucose; UACR: urinary albumin-to-creatinine ratio; sCr: serum creatinine; BUN: serum urea nitrogen; eGFR: glomerular filtration rate; HGB: hemoglobin; WBC: white blood cell; PLT: platelet; TC: total cholesterol; TG: triglycerides; ALB: serum albumin; UA: uric acid; CRP: C-reactive protein.

In the univariate analyses, we found that female sex (HR: 1.360, 95% CI: 1.000, 1.850, *p* = 0.048), diabetes duration (HR: 1.050, 95% CI: 1.010, 1.080, *p* = 0.004), higher HbA1C levels (HR: 1.110, 95% CI: 1.010, 1.230, *p* = 0.037), higher serum CRP levels (HR: 1.040, 95% CI: 1.000, 1.090, *p* < 0.001), and lower ABI (HR: 0.988, 95% CI: 0.983, 0.994, *p* < 0.001) were associated with ESKD events (Table 2, Figures 3 and 4). After adjustment for age, sex, smoking, alcohol consumption, duration of DM, use of statins, use of insulin, use of hypoglycemic drugs, use of angiotensin-converting enzyme inhibitor or angiotensin receptor blocker, HGB, HbA1C, eGFR, UACR, UA, ALB, triglycerides, TC, 24-h proteinuria, and CRP, the association of ABI and ESKD events did not change markedly(HR: 0.991, 95% CI: 0.985, 0.997, *p* = 0.004). Furthermore, after stratifying the ABI into three groups according to the upper and lower limits of its normal range and following adjustments for the above-mentioned confounding factors in the similar model, compared with the ABI normal range group, the ABI < 0.9 group remained a greater risk of ESKD events (HR: 1.758, 95% CI: 1.243, 2.487, *p* = 0.001; Table 3).

**Table 2.** Result of the univariate Cox proportional hazard model for associations of baseline clinical and laboratory parameters with ESRD in patients with advanced-stage diabetic kidney disease.

Variables	HR (95%CI)	*p*
Age (years)	1.001 (0.988–1.013)	0.899
Gender: Female vs Male	1.360 (1.000–1.850)	0.048*
Diabetic duration(years)	1.050 (1.010–1.080)	0.004*
Smoking	1.300 (0.920–1.840)	0.142
Drinking alcohol	0.870 (0.560–1.350)	0.534
BMI ( (kg/m^2^)	0.993 (0.953–1.033)	0.735
Insulin	1.080 (0.790–1.470)	0.633
Hypoglycemic drugs	1.350 (0.880–2.070)	0.163
ACEI or ARB	1.210 (0.890–1.640)	0.230
Statin	1.100 (0.810–1.500)	0.538
Aspirin	0.920 (0.680–1.250)	0.603
SBP (mmhg)	1.002 (0.994–1.009)	0.662
DBP (mmhg)	0.995 (0.982–1.007)	0.434
HbA1C (%)	1.110 (1.010–1.230)	0.037*
Glu (mmol/L)	0.930 (0.870–1.010)	0.080
24 hours proteinuria (mg)	1.000 (0.999–1.0001)	0.684
UACR (mg/g)	1.030 (0.890–1.190)	0.684
sCR (umol/L)	0.995 (0.989–1.001)	0.150
BUN (mmol/L)	0.990 (0.950–1.030)	0.602
eGFR(mL/(min·1. 73 m^2^ ))	1.004 (0.975–1.030)	0.771
HGB (g/L)	0.992 (0.974–1.009)	0.383
WBC (10°9/L)	1.030 (0.990–1.080)	0.162
PLT (10°9/L)	1.001 (0.998–1.003)	0.480
TC (mmol/L)	1.010 (0.910–1.130)	0.813
TG (mmol/L)	1.020 (0.940–1.110)	0.635
ALB (g/L)	0.980 (0.960–1.020)	0.331
UA (mmol/L)	1.000 (0.999–1.001)	0.635
CRP (mg/L)	1.040 (1.000–1.090)	0.047*
ABI (%)	0.988 (0.983–0.994)	<0.001*
0.9 ≤ ABI ≤ 1.3	Reference	
ABI < 0.9	2.073 (1.504–2.846)	<0.001*
ABI > 1.3	1.310 (0.740–2.316)	0.354

Abbreviations: HR: hazard ratio; 95% CI: 95% confidence interval; BMI: body mass index; ACEI: angiotensin-converting enzyme inhibitor; ARB: angiotensin receptor blocker; SBP: systolic blood pressure; DBP: diastolic blood pressure; HbA1C: glycated hemoglobin; Glu: serum glucose; UACR: urinary albumin-to-creatinine ratio; sCr: serum creatinine; BUN: serum urea nitrogen; eGFR: glomerular filtration rate; HGB: hemoglobin; WBC: white blood cell; PLT: platelet; TC: total cholesterol; TG: triglycerides; ALB: serum albumin; UA: uric acid; CRP: C-reactive protein; ABI: ankle brachial index. *p < 0.05.

**Table 3.** Associations between continuous and stratified ABI and ES**K**D.

	ES**K**D							
	Model 1		Model 2		Model 3		Model 4	
Variable	HR (95%CI)	*p*	HR (95%CI)	*p*	HR (95%CI)	*P*	HR (95%CI)	*p*
As continuous variable(%)								
ABI	0.988 (0.983–0.994)	<0.001	0.990 (0.985–0.996)	0.001	0.991 (0.985–0.997)	0.004	0.991 (0.985–0.997)	0.004
As stratification variable								
0.9 ≤ ABI ≤ 1.3	Reference							
ABI < 0.9	2.073 (1.504–2.846)	<0.001	1.853 (1.324–2.594)	<0.001	1.760 (1.251–2.475)	0.001	1.758 (1.243–2.487)	0.001
ABI > 1.3	1.310 (0.740–2.316)	0.354	1.337 (0.747–2.3869)	0.332	1.265 (0.707–2.261)	0.428	1.243 (0.691–2.236)	0.467

Model 1: crude; Model 2: adjusted for age, sex, smoking, drinking alcohol, and diabetic duration; Model 3: Model 2 plus statins, insulin, hypoglycemic drugs, ACEI or ARB, HbA1C, eGFR, and UACR; Model 4: Model 3 plus SBP, UA, ALB, TG, TC, HGB, 24 h proteinuria, and CRP.

ABI: ankle brachial index; ACEI: angiotensin-converting enzyme inhibitor; ARB: angiotensin receptor blocker; HbA1C: glycated hemoglobin; eGRF: glomerular filtration rate; UACR: urinary albumin-to-creatinine ratio; SBP: systolic blood pressure; UA: uric acid; ALB: serum albumin; HGB: hemoglobin; TC: total cholesterol; TG: triglycerides; CRP: C-reactive protein.

## Discussion

This study identified an association between a lower baseline ABI and ESKD in patients with advanced-stage diabetic kidney disease and found that individuals with ABI < 0.9 had a significantly higher rate of ESKD events, accounting for 42.5%. Patients with an ABI < 0.9 had an increased risk of ESKD compared to that in those with a normal ABI, indicating that patients with advanced-stage diabetic kidney disease with a lower baseline ABI, which is associated with arterial disease in the lower extremities, are at higher risk of developing ESKD. This is the first study to evaluate the precise role of a low ABI in the progression to ESKD in patients with advanced-stage diabetic kidney disease.

Diabetic nephropathy is a common microvascular complication of DM and is associated with macrovascular disease [20]. As kidney microvascular disease progresses, macrovascular sclerosis also progresses [21]. The correlation between the ABI and microvascular complications in patients with DM has been widely studied [6]. Previous studies have suggested that the ABI can be used to evaluate pathological changes in patients with diabetic nephropathy [9]. Patients with CKD are especially prone to complications of atherosclerosis, PAD, calcification of arterial walls, and increased arterial stiffness [22]. The ABI is used to detect PAD and is a marker of generalized atherosclerosis. The association between a low ABI and cardiovascular disease events has been well established [23], and several studies have reported an association between a low ABI and deteriorated kidney function [14,15,24]. The results of these previous studies indicate that a low ABI predicts future kidney function decline in the general population [14] and in patients with DM [9]. An ABI < 0.9 is associated with a greater risk of early-stage CKD after adjusting for traditional CKD risk factors in patients with DM, independent of albuminuria [15]. The results of the current study are consistent with previously reported results and indicate that a lower baseline ABI is independently associated with ESKD in patients with advanced-stage diabetic kidney disease, suggesting a close relationship between PAD and ESKD. However, the mechanisms of these relationships are complicated. One mechanism may be the kidney manifestation of systemic arteriosclerosis. Atherosclerosis can promote abnormal kidney function [25]. A recent paper from Subramanian at al. found that absent or diminished pedal pulses reflecting atherosclerosis correlated with eGFR decline in diabetics [26]. Those finding integrate with and support our results. In our study, patients with a low ABI had a higher SBP than those with a normal or high ABI. Arteriosclerosis may result in the transmission of an increased SBP to the glomerular capillaries, exacerbating glomerular hypertension, which is the main determinant of progressive kidney injury [27,28].

CRP, an inflammatory marker, may be independently associated with an abnormal ABI [23,29]. Consistent with that in previous studies, patients in the lower ABI group had a higher serum CRP level than patients in the normal ABI group in this study. These results indicate that inflammation may be a potential underlying mechanism of a low ABI. Inflammation may exacerbate kidney function, leading to ESKD in patients with advanced-stage diabetic kidney disease and is associated with microvascular and macrovascular complications in patients with DM [11,30]. Prolonged inflammation may result in aberrant adenosinergic signaling, which sustains inflammasome activation and worsens fibrotic reactions in target tissues within the kidney [31]. Inflammation and atherosclerosis interact and promote one another in patients with CKD [32], forming a cycle [33] that leads to the deterioration of kidney function.

An important methodological advantage of the current study is the analyses being conducted after the adjustment for renin-angiotensin system inhibitors, which have been shown to reduce proteinuria and protect kidney function [34]. However, this study is not without limitations. Firstly, markers of oxidative stress and other inflammatory markers, such as superoxide dismutase, malonaldehyde, interleukin 6, and tumor necrosis factor α, were not measured at baseline. Secondly, data regarding the use of mineralocorticoid receptor antagonist medications were not available, which may be related to the prognosis of kidney function and we did not record the events of myocardial infarction and stroke, or other cardiovascular diseases that are associated with arteriosclerosis. Thirdly, the study population had been in a poorer glycemic control and the HbA1c level during the follow-up period was not available, which may also interfere the outcomes. Lastly, this single-center, retrospective study was conducted using data from a database. Therefore, bias cannot be ruled out.

In conclusion, lower-extremity arterial disease, defined as a baseline ABI < 0.9, may be associated with ESKD in patients with advanced-stage diabetic kidney disease. Potential mechanisms of this association include atherosclerosis, inflammation, and subsequent deterioration of kidney function. Improving atherosclerosis and the inflammatory status in patients with advanced-stage diabetic kidney disease may provide clinical benefits in the long-term kidney survival of patients with residual kidney function. However, prospective studies with larger sample sizes are needed.

## Data Availability

The datasets used and/or analyzed during the current study are available from the corresponding author on reasonable request.
